# Paracrine SPARC signaling dysregulates alveolar epithelial barrier integrity and function in lung fibrosis

**DOI:** 10.1038/s41420-020-0289-9

**Published:** 2020-06-30

**Authors:** Franco Conforti, Robert Ridley, Christopher Brereton, Aiman Alzetani, Benjamin Johnson, Ben G. Marshall, Sophie V. Fletcher, Christian H. Ottensmeier, Luca Richeldi, Paul Skipp, Yihua Wang, Mark G. Jones, Donna E. Davies

**Affiliations:** 1 Clinical and Experimental Sciences, Faculty of Medicine, University of Southampton, Southampton, SO16 6YD UK; 2 NIHR Southampton Biomedical Research Centre, University Hospital Southampton, Southampton, SO16 6YD UK; 3 Department of Thoracic Surgery, University Hospital Southampton, Southampton, SO16 6YD UK; 4 University Hospital Southampton, Southampton, SO16 6YD UK; 5 Cancer Sciences & NIHR and CRUK Experimental Cancer Sciences Unit, University of Southampton, Southampton, SO16 6YD UK; 6 Unità Operativa Complessa di Pneumologia, Università Cattolica del Sacro Cuore, Fondazione Policlinico A. Gemelli, Rome, Italy; 7 Centre for Proteomic Research, Institute for Life Sciences University of Southampton, Southampton, SO17 1BJ UK; 8 Institute for Life Sciences, University of Southampton, Southampton, SO17 1BJ UK; 9 Biological Sciences, Faculty of Natural and Environmental Sciences, University of Southampton, Southampton, SO17 1BJ UK

**Keywords:** Extracellular signalling molecules, Experimental models of disease

## Abstract

Idiopathic pulmonary fibrosis (IPF) is a chronic scarring disease in which aging, environmental exposure(s) and genetic susceptibility have been implicated in disease pathogenesis, however, the causes and mechanisms of the progressive fibrotic cascade are still poorly understood. As epithelial–mesenchymal interactions are essential for normal wound healing, through human 2D and 3D in vitro studies, we tested the hypothesis that IPF fibroblasts (IPFFs) dysregulate alveolar epithelial homeostasis. Conditioned media from IPFFs exaggerated the wound-healing response of primary human Type II alveolar epithelial cells (AECs). Furthermore, AECs co-cultured with IPFFs exhibited irregular epithelialization compared with those co-cultured with control fibroblasts (NHLFs) or AECs alone, suggesting that epithelial homeostasis is dysregulated in IPF as a consequence of the abnormal secretory phenotype of IPFFs. Secretome analysis of IPFF conditioned media and functional studies identified the matricellular protein, SPARC, as a key mediator in the epithelial–mesenchymal paracrine signaling, with increased secretion of SPARC by IPFFs promoting persistent activation of alveolar epithelium via an integrin/focal adhesion/cellular-junction axis resulting in disruption of epithelial barrier integrity and increased macromolecular permeability. These findings suggest that in IPF fibroblast paracrine signaling promotes persistent alveolar epithelial activation, so preventing normal epithelial repair responses and restoration of tissue homeostasis. Furthermore, they identify SPARC-mediated paracrine signaling as a potential therapeutic target to promote the restoration of lung epithelial homoestasis in IPF patients.

## Introduction

Idiopathic pulmonary fibrosis (IPF) is a chronic progressive lung disease with limited responsiveness to current therapies and a prognosis similar to lung cancer^1,2^. The current paradigm for IPF pathogenesis postulates that repetitive alveolar epithelial injuries lead to aberrant fibroblast proliferation and formation of the fibroblastic foci which results in exaggerated deposition of extracellular matrix (ECM), destruction of the lung parenchymal architecture and marked impairment of gas exchange^1,3,4^. Although the pathogenesis of lung fibrosis is viewed as a result of both genetic and environmental risk factors, little is known about the underlying mechanisms driving abnormal injury/repair responses in IPF. The complex interactions between the persistent injured epithelium and the abnormal activated fibroblasts could to be one of the main factors responsible for disease progression.

The functional interactions between epithelial cells and mesenchymal cells, as well as the ECM which plays a central role in the control of tissue homeostasis, are described through the concept of epithelial–mesenchymal trophic unit (EMTU)^5^. Increasing evidence suggests that alveolar epithelial damage and resulting abnormal epithelial–mesenchymal crosstalk, and therefore dysregulation of the lung EMTU, may also contribute to the aberrant wound-healing response observed in the lungs of IPF patients^6–12^. During a normal wound-healing process, both epithelial and mesenchymal cells release soluble factors that affect the behavior of resident and nearby infiltrating cells^5,13^, while in vitro studies suggest that in comparison with control normal human lung-derived fibroblasts (NHLFs), IPF lung-derived fibroblasts (IPFFs) make less hepatocyte growth factor (HGF) and prostaglandin E_2_ (PGE2), both critical factors involved in epithelial repair and suppression of fibrosis^11,14^ while they exhibit increased IL6 stimulated proliferation and reduced apoptosis^15,16^.

Although, dysregulation of epithelial–mesenchymal crosstalk in IPF is likely to be a key determinant of progressive fibrosis, little is understood regarding direct cross talk between fibroblasts and epithelial cell in IPF. Since cell–cell and cell–ECM interactions direct cell proliferation, migration and differentiation in the synchronization of physiological events like inflammation, angiogenesis, epithelialization, and tissue remodeling^17,18^, it is likely that miscommunication between epithelial and mesenchymal cells due to the abnormal secretory phenotype of IPFFs plays an important role in the development and progression of the disease.

We hypothesized that, in IPF, parenchymal fibroblasts alter epithelial behavior and that this dysregulates the alveolar EMTU nexus and promotes persistent alveolar epithelial activation, so preventing normal epithelial repair responses and restoration of tissue homeostasis.

## Results

### Paracrine signaling from IPF fibroblasts augments the epithelial wound repair response

**Fig. 1:**
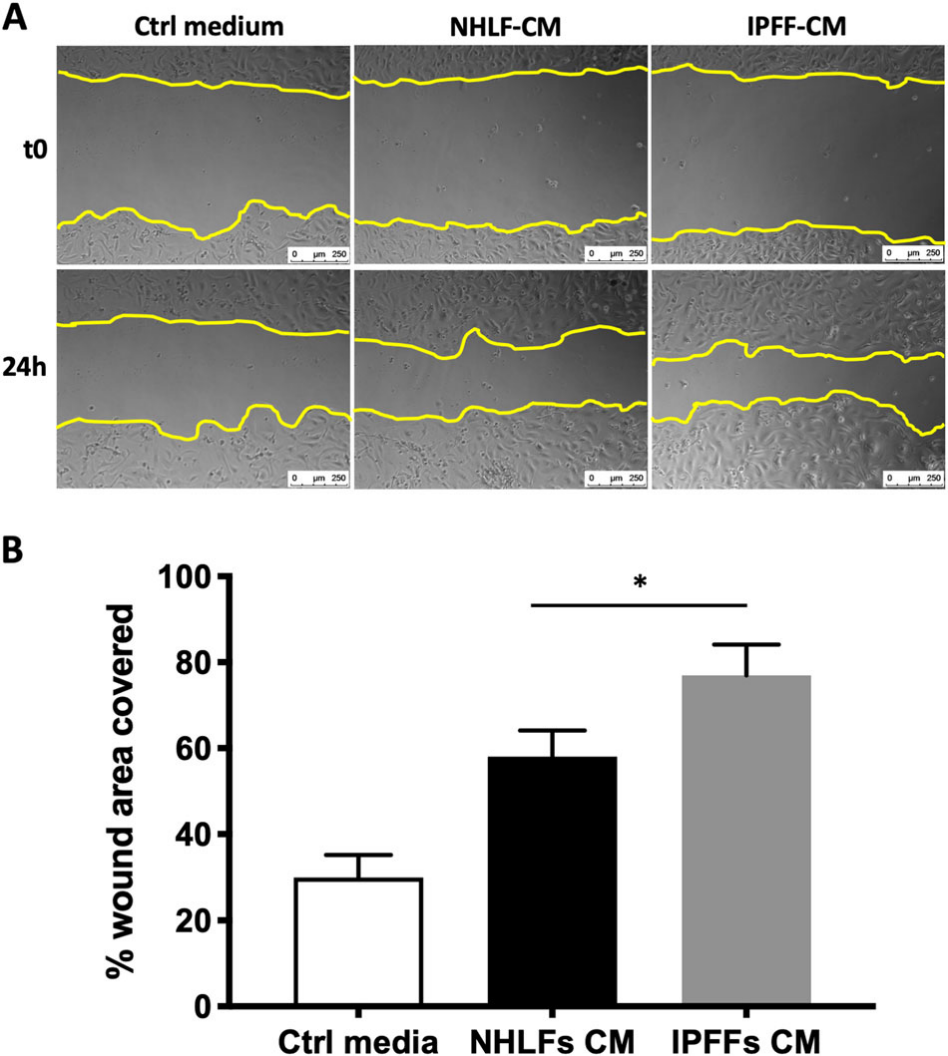
The effect of fibroblast conditioned media on repair of primary AEC monolayers following a scratch wound. **a** Representative images of scratch wound assay at time 0 (*t*0) and 24 h using primary AECs treated without or with IPFF-CM or NHLF-CM and **b** wound area quantification analyzed using the FIJI quantification tool from ImageJ. Data presented as mean ± SD, *n* = 4. *p < 0.05 using non-parametric *t*-test.

The in vitro scratch wound-healing assay mimics cell migration during wound healing in vivo, enabling the investigation of cell–cell interactions^19^. To assess the effect of fibroblast-derived secreted factors and therefore the paracrine signaling on respiratory epithelial cells during injury/repair of the epithelium, we performed a scratch wound-healing assay on confluent primary human Type II alveolar epithelial cells (AECs) in the absence or presence of conditioned media (CM) from NHLF (NHLF-CM) or IPFF (IPFF-CM). Compared with AECs treated with standard culture media, CM from lung fibroblasts increased AEC migration so accelerating the wound repair response, and this was significantly greater with CM from IPFFs compared with NHLFs (Fig. 1a, b). These findings suggest that secreted factors from IPFFs promote an exaggerated wound repair response.

### IPF fibroblasts show increased levels of the matricellular protein SPARC

**Fig. 2:**
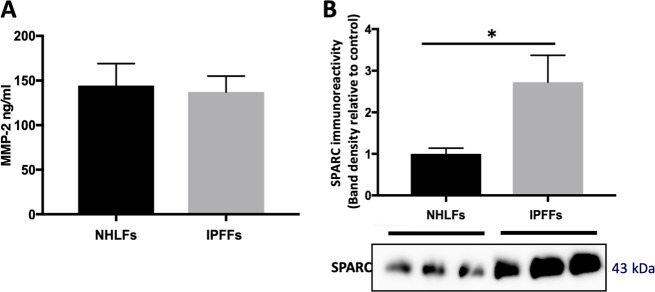
Matricellular proteins levels detected in the conditioned media from IPFFs and lung alveolar cells. Fibroblasts were seeded at 10,500 cells/cm^2^ and allowed to condition the media for 48 h. Equal volumes of cell-free supernatants were then analyzed for MMP2 or SPARC protein levels. **a** MMP2 levels detected by Luminex multiplex assay of IPFF-CM and NHLF-CM. Data presented as mean ± SD (*n* = 3). Statistical significance tested by ANOVA. **b** Western blotting analysis of SPARC protein levels in conditioned media from IPFF-CM and NHLF-CM, *n* = 3.

In order to investigate the secreted factors from IPFFs that may promote an exaggerated wound repair response, we analyzed the secretome of IPFFs detecting 433 proteins; among the most abundant proteins we identified two possible candidates responsible for the wound-healing response (Table S1): the metalloprotease MMP2 (matrix metallopeptidase 2) and the matricellular protein SPARC (secreted protein acidic and rich in cysteine). Comparing their levels in NHLF-CM and IPFF-CM, we found no difference in levels of MMP2 (Fig. 2a) while SPARC was significantly higher in CM from IPFFs (Figs. 2b-S1a). Furthermore, while SPARC was secreted at high levels from IPFFs, it was low or absent in CM of AECs or the H441 lung epithelial cell line (Fig. S1c). Therefore in IPF, the lung fibroblasts are likely to be the main source of this matricellular protein.

### Fibroblast-secreted SPARC regulates alveolar epithelial cell migration

**Fig. 3:**
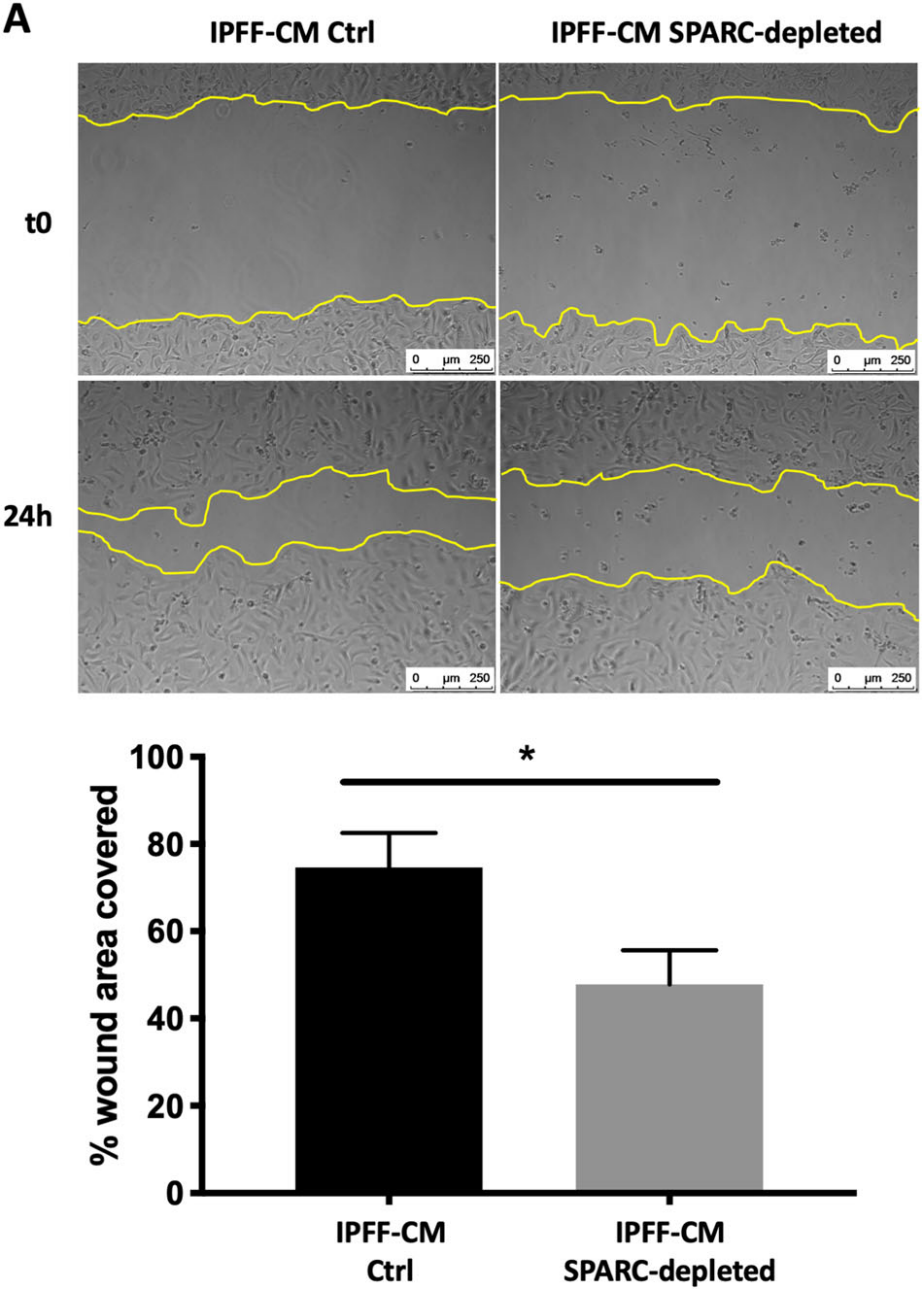
The effect of SPARC depletion on scratch wound repair responses of primary AECs treated with conditioned media from IPFFs. **a** Representative images of scratch wounds at time 0 (*t*0) and 24 h using primary AECs treated with IPFF-CM control (Ctrl) or IPFF-CM SPARC-depleted. The wound area was quantified using the FIJI quantification tool (ImageJ). Data presented as mean ± SD, *n* = 3. *p < 0.05 using non-parametric *t*-test.

To investigate the influence of fibroblast-secreted SPARC upon AEC wound repair we depleted IPFF-CM of SPARC using siRNA (Fig. S1b), and then applied the CM to the AEC during a scratch wound assay. This identified that SPARC depletion significantly reduced the ability of IPFF-CM to promote AEC wound healing (Fig. 3a). In contrast SPARC depletion did not affect the ability of TGFβ_1_ to induce differentiation of the fibroblasts into myofibroblasts (Fig. S1b). Thus, SPARC secretion is significantly increased in IPFFs and acts as a key paracrine stimulus that increases AECs migration.

### SPARC enhances alveolar cell migration via integrin signaling

**Fig. 4:**
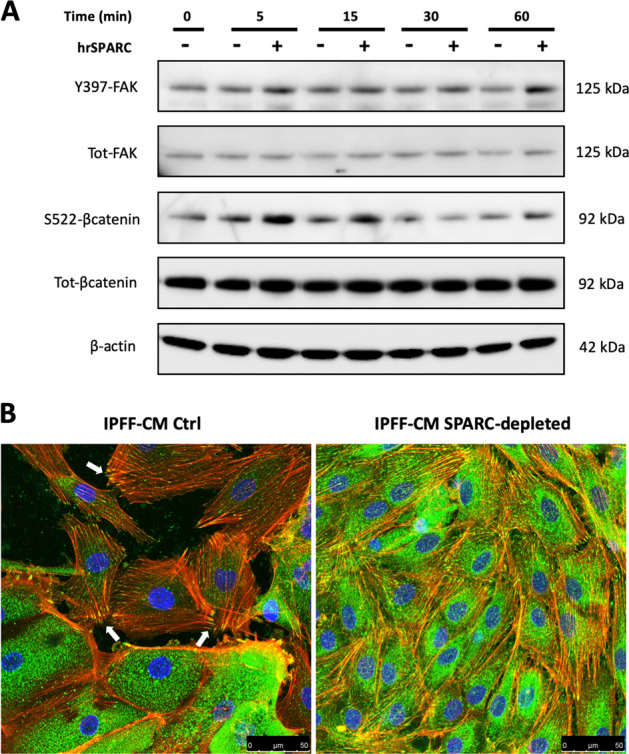
The effect of SPARC on focal adhesions in AECs monolayers. **a** Western blot showing Y397 phosphorylation of FAK and S522 phosphorylation of β-catenin during time course treatment of AECs without (Ctrl) and with (+SPARC) human recombinant SPARC (hrSPARC). **b** Immunofluorescence images of AECs after treatment with IPFF-CM control (Ctrl) or IPFF-CM SPARC-depleted; cells were fixed and stained for paxillin (green) to identify focal adhesions (indicated by white arrows), TRICT-phalloidin (red) to identify F-actin and DAPI (blue) was used to stain nuclei.

In order to investigate the mechanism whereby SPARC infleunces AEC migration, pathway analysis was performed on proteins in the IPFF secretome (Table 1). This identified pathways including focal adhesion and receptor interactions with the ECM. Since focal adhesions consist of large aggregates of transmembrane receptors, integrins, that interact with the ECM^20,21^ and the focal-adhesion kinase (FAK) is a key cytoplasmic tyrosine kinase involved in integrin-mediated cell migration^22^, we investigated the effect of SPARC upon FAK activation. Treatment of AECs with recombinant human SPARC for up to 1 h identified increased Y397 phosphorylation of FAK (Fig. 4a) consistent with integrin activation. In parallel with FAK phosphorylation, we detected the specific S552 phosphorylation of β-catenin (Fig. 4a) that has been reported to induce its dissociation from adherens junctions where it interacts with E-cadherin to regulate cell–cell adherens junction formation^23,24^. Furthermore, treatment of AECs with IPFF-CM resulted in formation of large focal adhesions that co-localized with lamellipodia/filopodia connecting to the F-actin network (Fig. 4b), consistent with enhanced cell migration in response to Y397 phosphorylation of FAK. These changes correlated with disorganization of epithelial layer while SPARC depletion was able to reestablish a uniform and contiguous epithelial cell layer that is required for a functional epithelial barrier.

**Table 1:** Kyoto Encyclopedia of Genes and Genomes (KEGG) pathway analysis of the top 100 most abundant protein detected in the conditioned media of IPFFs.

	KEGG pathways		
Pathway	Description	Count in gene set	False discovery rate
hsa04512	ECM-receptor interaction	13 of 81	1.68e−13
hsa04510	Focal adhesion	16 of 197	7.06e−13
hsa04610	Complement and coagulation cascades	9 of 78	2.23e−08
hsa04974	Protein digestion and absorption	9 of 90	5.37e−08
hsa05146	Amoebiasis	9 of 94	6.13e−08
hsa04151	PI3K-Akt signaling pathway	13 of 348	7.00e−07
hsa05165	Human papillomavirus infection	12 of 317	1.82e−06
hsa05205	Proteoglycans in cancer	9 of 195	1.41e−05
hsa04933	AGE-RAGE signaling pathway in diabetic complications	7 of 98	1.41e−05
hsa05150	Staphylococcus aureus infection	5 of 51	0.00011
hsa05322	Systemic lupus erythematosus	6 of 94	0.00013
hsa05133	Pertussis	5 of 74	0.00049
hsa04066	HIF-1 signaling pathway	5 of 98	0.0016
hsa04670	Leukocyte transendothelial migration	5 of 112	0.0027
hsa04926	Relaxin signaling pathway	5 of 130	0.0048
hsa05410	Hypertrophic cardiomyopathy (HCM)	4 of 81	0.0067
hsa05132	Salmonella infection	4 of 84	0.0072
hsa05134	Legionellosis	3 of 54	0.0195
hsa04611	Platelet activation	4 of 123	0.0244
hsa04810	Regulation of actin cytoskeleton	5 of 205	0.0252
hsa04520	Adherens junction	3 of 71	0.0349
hsa04145	Phagosome	4 of 145	0.0370
hsa05206	MicroRNAs in cancer	4 of 149	0.0388

### Increased levels of fibroblast-secreted SPARC dysregulate alveolar epithelial junctional organization and barrier integrity

**Fig. 5:**
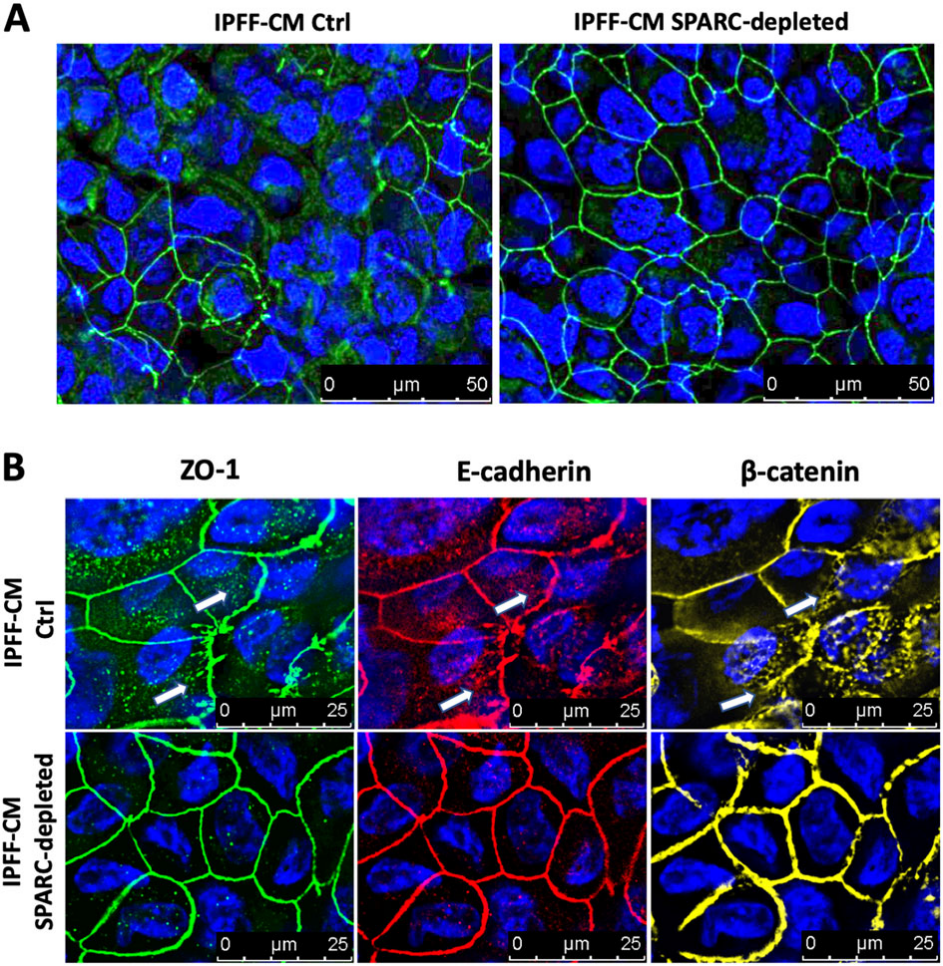
The effect of SPARC depletion from IPFF-CM on epithelial adhesion junctions. **a** H441 lung epithelial cells were treated with IPFF-CM control (Ctrl) or IPFF-CM SPARC-depleted; cells were then fixed and stained for TJs or AJs and imaged using epifluorescence microscopy. Representative immunofluorescence images of TJs (ZO-1 in green) and nuclei (DAPI in blue). **b** Higher magnification immunofluorescence images showing ZO-1, E-cadherin, and β-catenin localization; white arrows indicating cytosolic redistribution. Representative immunofluorescence images of TJs (ZO-1 in green), AJs (E-cadherin in red), AJs (β-catenin in yellow), and nuclei (DAPI in blue).

Given the ability of IPFF-CM to augment the healing response in the scratch wound assays, we next investigated whether it also affected barrier formation since the integrity of epithelial barrier is essential for the maintenance of tissue homeostasis where it is controlled by specialized cell adhesion complexes, especially tight junctions (TJs) that control paracellular permeability^25–27^. When confluent lung epithelial cells were treated with IPFF-CM we identified disorganization of TJs, as detected by ZO-1 immunofluorescent staining (Fig. 5a) while SPARC depletion prevented this effect. In addition, we observed that high levels of SPARC present in IPFF-CM also disrupted adherens junctions (AJs) as detected by E-cadherin and β-catenin immunofluorescent staining which showed partial delocalization into the cytosol (Fig. 5a, b).

**Fig. 6:**
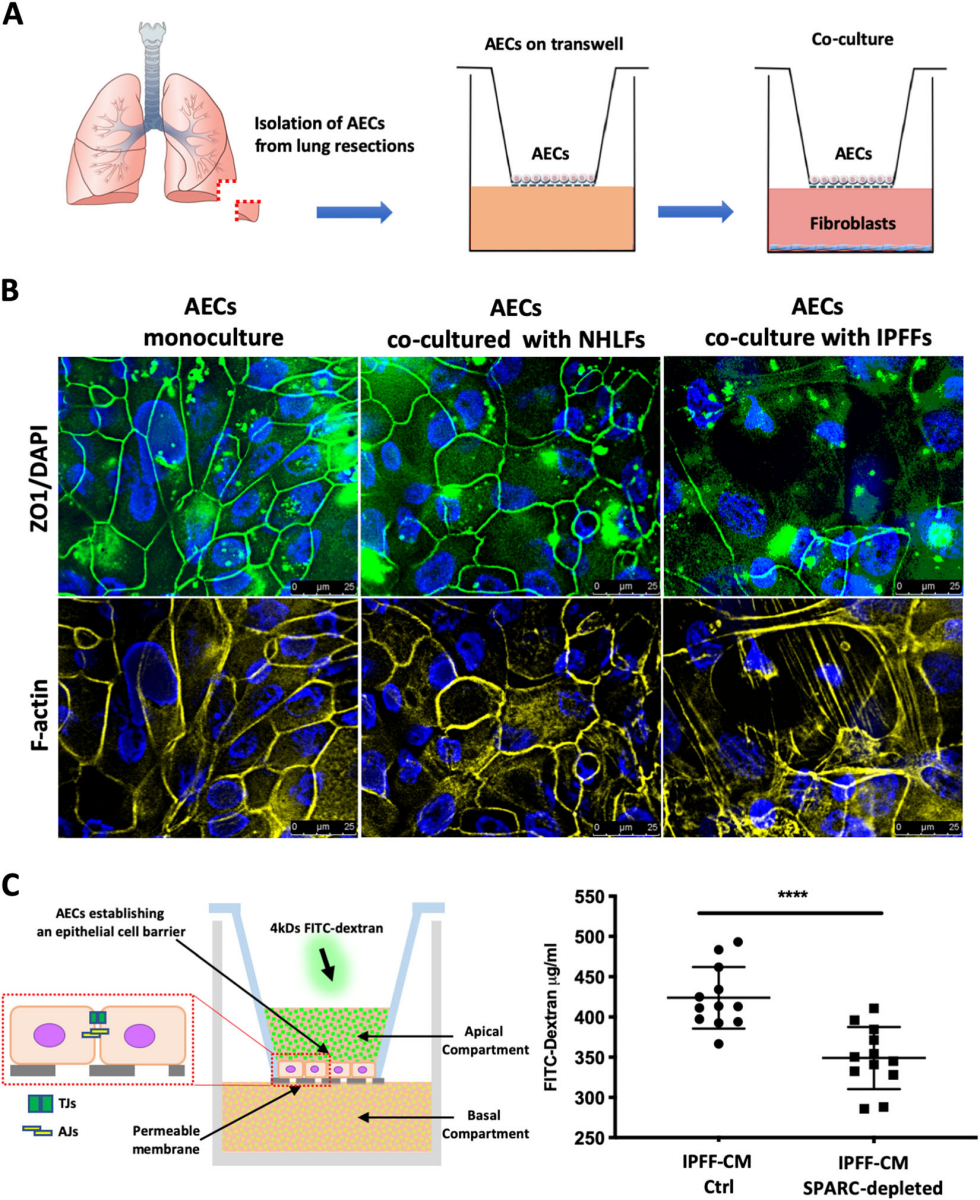
Effect of IPFF-derived SPARC on macromolecular permeability of AECs. **a** Schematic representation of AECs isolation and setup of the Transwell AEC-fibroblast co-culture model. **b** Fluorescence images for ZO-1 immunostaining in AECs after co-culture with IPFFs compared with NHLFs. Representative immunofluorescence images of TJs (ZO-1 in green), F-actin (TRICT-phalloidin in yellow), and nuclei (DAPI in blue). **c** Measurement of paracellular permeability of AECs in Transwell culture after pre-treatment for 6 days with IPFF-CM control (Ctrl) or IPFF-CM SPARC-depleted. The figure shows permeability of the AEC cell layer to FITC-dextran (4 kDa) measured by its ability to pass from the apical compartment to the basolateral compartment.

As the application of IPFF-CM on AECs is a unidirectional stimulus that only partially recapitulates paracrine signaling between lung fibroblast and alveolar epithelial cells, we established an ex vivo co-culture model of human primary FFs with primary AECs (Fig. 6a). This co-culture system allows the cells to share the same microenvironment to better mimic cell–cell paracrine interactions that may occur in in vivo. Consistent with our findings when using IPFF-CM, we detected disorganization of TJs in AECs co-cultured with IPFFs compared with AECs co-cultured with NHLFs (Fig. 6b). Under the same conditions, AEC number was not significantly affected by the presence of NHLFs or IPFFs (Fig. S2).

A functional epithelial barrier is characterized by the establishment of a continuous layer of polarized epithelial cells that control the paracellular passage of ions and molecules^28^. Since SPARC alters the junctional organization that is critical for maintaining cell polarization, we investigated if the high levels of SPARC secreted by IPFFs affected AEC paracellular permeability to macromolecules (Fig. 6c). We detected a significant increase in paracellular permeability in AECs treated with IPFF-CM compared with AECs treated with IPFF-CM depleted of SPARC using siRNA (Fig. 6c). Together these results suggest that the increased secretion of SPARC by IPFFs affect the integrity of the alveolar epithelium by altering the organization of cellular junctions and preventing the reestablishment of tissue homeostasis after lung injury.

## Discussion and conclusions

IPF fibroblasts have been reported to secrete an array of factors including an abnormal ECM that stimulates fibroblast activation and proliferation^15,16,29–32^. These studies indicate that the distinctive secretory phenotype of IPFFs plays an important role in the development and progression of the disease, however, investigations of their effect on alveolar epithelial cells are limited. In our study we tested the effect of the secretome from primary IPF lung fibroblasts on primary human Type II alveolar epithelial cells and dissected one of the key molecular pathways involved in epithelial–mesenchymal cross talk. We identified the matricellular protein SPARC is highly secreted by IPFFs and we have demonstrated that it is a key mediator involved in the abnormal paracrine cross talk between the AECs and lung fibroblasts isolated from patients affected by IPF. We showed that SPARC is a strong promoter of alveolar epithelial wound healing in scratch wound assays. While this was suggestive of a beneficial effect, we demonstrated that it maintained a persistent wound-healing phenotype of the alveolar epithelium and prevented the reestablishment of a functional alveolar barrier. Together these studies identify that in lung fibrosis increased lung fibroblast SPARC secretion signaling dysregulates alveolar epithelial barrier integrity and function so preventing normal epithelial repair responses and restoration of tissue homeostasis.

The ECM is a major determinant of the tissue microenvironment able to modulate cell proliferation, adhesion, migration, and differentiation. Since fibroblasts are the main producers of ECM, their biosynthetic activity is likely to affect homeostasis within the lung microenvironment^33–35^. SPARC is a highly conserved matricellular protein that modulates interactions between cells and the extracellular environment. Different studies have shown a role for SPARC in the regulation of MMPs, ECM assembly, collagen formation/deposition, and growth factors signaling^36^. Its levels are elevated in tissues with high cell turnover, in embryonic development and in response to injury but it is reduced in normal adult tissues suggesting its key role in tissue regeneration and repair^37,38^. In the lung, SPARC has been described to be involved in cancer development, in chronic airway diseases and pulmonary fibrosis^39–43^ where it controls tissue remodeling, cell proliferation and migration. In lung fibrosis SPARC has been shown suppress apoptosis of IPF lung fibroblasts^44^ and being a down-stream effector of the profibrotic cytokine TGF-β^43,45^. Moreover, different in vivo studies in mouse models of bleomicyn-induced lung fibrosis, SPARC-null mice show attenuation of fibrotic changes mainly in terms of fibroblast collagen secretion and accumulation^42,46,47^. Consistent with a previous report^44^, we found that SPARC is upregulated in lung fibroblasts isolated from IPF patients compared with lung fibroblasts isolated from age-matched non-IPF donors. Furthermore, we found that SPARC is prefentially expressed in IPF fibroblasts compared with AECs; these findings are consistent with recent single-cell RNA sequencing data assembled together in the IPF Cell Atlas server (http://www.ipfcellatlas.com) which show preferential expression of SPARC in stromal/mesenchymal cells compared with AECs and identify subpopulations within the stromal/mesenchymal cells with higher expression in IPF.

SPARC is highly expressed during development and in response to injury in order to stimulate an intermediate adhesive cell state characterized by reorganization of the focal adhesions that promote cell migration^48^. Likewise, in cancer cells, it has been shown to increase cell migration by modulating the activation of integrins within focal adhesions^49–51^. The integrin cell–matrix interaction also regulates the formation and stability of cell–cell junctions via protein kinases associated with focal adhesions, such as focal-adhesion kinase (FAK)^52–55^. An in vivo study on lung metastasis reported that genetic inhibition of FAK in endothelial cells prevented tumor cell extravasation from lung vessels and metastasis by preserving the integrity and functionality of the vascular barrier^56^. Here, we found that AECs challenged with human recombinant SPARC exhibited high levels of active Y397 phosphorylated focal-adhesion kinase (FAK) and S522 phosphorylated β-catenin that are both markers of integrin activation, leading to decreased cell adhesion and activation of cell migration. We also noted that even at baseline there was some evidence of FAK phosphorylation. Since cells within an epithelial layer exhibit motion, this will require the reorganization of focal adhesions involving cycles of phosphorylation and dephosphorylation of FAK.

The protein kinase FAK is not only a key regulator of integrin-mediated cell–matrix interaction, but it has also been reported to control the AJs. Following integrin engagement, FAK becomes auto-phosphorylated on Y397 and binds to Src kinase, increasing its activity in association with the disruption of E-cadherin dependent AJs^57,58^. In particular, it has been shown that during the establishment of cell–cell contact, there is an accumulation of E-cadherin–β-catenin–α-catenin complexes at adhesion sites in the plasma membrane due to the homotypic interactions between E-cadherins of adjacent cells, with a consequent reduction of its cytosolic fraction. In absence of cell–cell contact, E-cadherin–β-catenin complexes are rapidly redistributed from the membrane into the cytosol via endocytosis^59–61^. The establishment of AJs has been shown to be essential for the formation and localization of TJs that control epithelial polarization and paracellular permeability^62,63^. The activation of β1 integrins has been shown to be crucial for the intracellular relocation of the TJ proteins zona occludens 1 (ZO-1) and claudins during cellular repolarization^64^. In our investigation we found that IPFF-CM caused disorganization of both AJs and TJs in the AEC layer, with accumulation of E-cadherin, β-catenin, and ZO-1 in the cell cytosol, while depletion of SPARC restored the proper organization of both AJs and TJs in AEC layer. These findings were reinforced by the observations that when AECs were co-cultured with IPFFs, that secrete high level of SPARC, TJs were poorly formed compared with those seen when AECs were co-cultured with NHLFs that secrete much less SPARC.

While the AEC barrier must allow gas exchange between the inhaled air and the blood, it must also function as a physcial barrier that regulates the paracellular flux of ions and macromolecules. Moreover, it participates in the immune response defending the underlying tissues from inhaled pathogens, pollutants, and other noxious agents^65^. In an animal model of acute respiratory distress syndrome (ARDS), an injury to the AEC barrier resulted in severe alveolar edema and compromised lung function whereas damage to the endothelium alone was not sufficient to cause pulmonary edema^66^. The loss of AEC barrier integrity not only leads to increased edema into the interstitial and alveolar spaces; it also lead to infiltration and activation of local and/or circulating fibroblasts with deposition of provisional ECM in the alveoli^67^. Thus, restoring the AEC barrier as efficiently as possible after injury is crucial for normal function of the alveoli. As the establishment of cell–cell and cell–matrix adhesions are key aspects of collective cell movement and the reestablishment of paracellular barrier^17^, our identification of the SPARC/integrin/focal adhesion/cellular junction may be of clinical relevance in a tissue injury/repair scenario such as lung fibrosis. In response to lung damage, the type 2 AECs start proliferating, migrating and differentiating into the injured area to restore the functional respiratory epithelium^68^. The transmembrane integrins are the nexus between the ECM and the intracellular signaling that drive the cell movement while the apical-lateral boundaries of polarized epithelial cells are delineated by two main distinct intercellular junctions, the AJs and TJs^69^. The interplay between these cellular components is critical for collective cell migration and maintaining cellular polarization^70,71^. In the abnormal wound-healing setting of lung fibrosis, we have identified that the exaggerated secretion of SPARC by IPFFs has a negative impact on the proper resolution of the lung epithelium following injury by preventing the reestablishment of the functional pulmonary barrier. This may result in exposure of subepithelial cells to environmental insults leading to chronic tissue injury and disease progression. This concept is consistent with a recent immunohistochemical study proposing epithelial barrier alterations in IPF lung tissue^72^.

In conclusion, our study identifies a role for SPARC in perturbing epithelial barrier homeostasis in human lung fibrosis. Furthermore, it identifies SPARC-mediated paracrine signaling as a potential therapeutic target to promote the restoration of normal epithelium integrity so preventing the progression of progressive lung fibrosis.

## Materials and methods

### Cell culture and isolation

#### Human lung tissue sampling

All human lung experiments were approved by the Southampton and South West Hampshire and the Mid and South Buckinghamshire Local Research Ethics Committees, and all subjects gave written informed consent. Clinically indicated IPF lung biopsy tissue samples and non-fibrotic control tissue samples (macroscopically normal lung sampled remotely from a cancer site) were deemed surplus to clinical diagnostic requirements. All IPF samples were from patients subsequently receiving a multidisciplinary diagnosis of IPF according to international consensus guidelines^73^.

#### Primary fibroblast culture

Primary fibroblast cultures were established from lung parenchyma tissue of patients with IPF obtained by video-assisted thoracoscopic lung biopsy at University Hospital Southampton (*n* = 3) or normal lung parenchyma tissue (*n* = 3). Fibroblasts were cultured in Dulbecco’s modified Eagle’s medium (DMEM) supplemented with 10% fetal bovine serum (FBS), 50 units/ml penicillin, 50 μg/ml streptomycin, 2 mM L-glutamine, 1 mM sodium pyruvate, and 1x non-essential amino acids (DMEM/FBS) (Life Technologies, Paisley, UK). Fibroblasts were treated in the absence or presence of 5 ng/ml TGF-β1 (PeproTech, London, UK).

#### Primary alveolar type II (AECs) cell culture

Primary AECS culture were established from macroscopically normal regions of surgically resected lung parenchyma tissue in accord with the method described by Witherden et al.^74^. Briefly, the lung tissue was perfused with 0.9% sodium chloride solution and infused with 0.25% Trypsin solution (Sigma-Aldrich, Poole, UK) at 37 °C for 45 min. After trypsin digestion, the tissues were finely cut in the presence of newborn calf serum (NCS) and DNase (250 μg/ml), then cells were filtered by sequential passage through a 400-μm metal mesh and 40-μm nylon filter. The cells were re-suspended in DCCM-1 medium (Biological Industries Ltd, Kibbutz Beit-Haemek, Israel) supplemented with 1% penicillin, 1% streptomycin, and 1% L-glutamine, and incubated at 37 °C in a humidified incubator for 2 h in tissue culture flasks to allow differential adherence and removal of contaminating cells. The alveolar epithelial cells were re-suspended in fresh DCCM-1 supplemented with 10% NCS, 1% penicillin, 1% streptomycin and 1% L-glutamine and plated on collagen 1 (PureCol 5005-b, Advanced BioMatrix Inc, California, USA) coated 9-well plates at 60% density; after 72 h purity was tested by staining for alkaline phosphatase. For the co-culture model AECs were plated on apical side of Transwell® Clear inserts (Corning, VWR, Dublin, Ireland) containing polyethylene membranes with a pore size 0.4 μm. Before use, the membranes were coated with collagen Type 1 (PureCol 5005-b, Advanced BioMatrix Inc, California, USA). After 6 days the AECs culture inserts were transferred to fresh wells containing fibroblasts (seeded 48 h previously) as illustrated in Fig. 6a.

#### Lung epithelial cell line

NCl-H441 (American Type Culture Collection, HTB-174) cells were obtained from LGC Standards (Teddington, UK) and cultured at 37 °C in air supplemented with 5% CO_2_ in Gibco RPMI-1640 medium (Life Technologies, Paisley, UK) containing 10% fetal bovine serum (FBS), 1% sodium pyruvate, 100 U/mL penicillin, and 100 μg/mL streptomycin (all from Sigma-Aldrich, Dublin, Ireland).

### Macromolecular permeability assay

Primary AECs cells (*n* = 2 donors) were cultured on the apical side of Transwell® Clear inserts (Corning, VWR, Dublin, Ireland) until the formation of a confluent epithelial monolayer and treated with conditioned media (CM) of IPFFs (*n* = 3 patients) replete with, or depleted of, SPARC. After 6 days of CM treatment, 4 kDa dextran labeled with fluorescein isothiocyanate–dextran (FITC-dextran, 2 mg/ml) (Sigma-Aldrich, Dublin, Ireland) was added to the apical compartment of the tranwell culture and after 24 h the fluorescence signal from the FITC-dextran passed across the epithelial monolayer in the basal compartment was detected using a Fluoroskan plate reader Ascent FL (Thermofisher, Basingstoke, UK).

### Cell count and treatment

Primary AECs cells (*n* = 3) from transwell monoculture or co-culture with lung fibroblasts were fixed with 4% paraformaldehyde solution and nuclei stained with 4′,6-diamidino-2-phenylindole (DAPI). For each condition, 5 fields of cell nuclei were imaged using Leica DMI 6000B (Leica Microsystem, Milton Keynes, UK) and counted using ImageJ software. For the protein phosphorylation time course AECs after reaching 70–80% cell confluence were starved 2 h in DCCM-1 supplemented with 1% penicillin, 1% streptomycin, and 1% l-glutamine, and treated with recombinant human SPARC 2 μg/ml at the indicated time points.

### Secretome analysis of primary lung fibroblasts using mass spectrometry

Label-free Mass Spectrometry analysis (LC-MS^E^) was performed on primary lung fibroblasts conditioned media in accord with our previous described method^12^. The proteomic data were analyzed using STRING protein network server.

### Time lapse and scratch wound-healing assay

A scratch wound-healing assay was performed on a confluent monolayer of primary alveolar epithelial cells cultured on multi-well cell culture plate in nutrient depleted media. A cell-free area was created by wounding the confluent cell monolayer with a pipette tip and images where acquired by time-lapse microscopy over a 24 h period using Leica DMI 6000B (Leica Microsystem, Milton Keynes, UK). Wound area was measured using image analysis with FIJI ImageJ software.

### Luminex multiplex assay

Magnetic Luminex® Performance Assays were performed to measure MMP proteins in IPFF-CM and NHLF-CM in accord with manufacturer’s instructions.

### Cell transfection

Short interfering RNA oligonucleotides for SPARC (SPARC ON-TARGETplus SPARC siRNA) and non-targeting control siRNA (ON-TARGETplus Non-targeting Pool) were obtained from Dharmacon GE Medical Systems Ltd, Buckinghamshire, UK, and used on primary lung fibroblasts according to the manufacturer’s instructions. After 48 h of gene silencing, primary lung fibroblasts were incubated in DMEM supplemented with 50 units/ml penicillin, 50 μg/ml streptomycin, 2 mM L-glutamine, 1 mM sodium pyruvate, and 1x non-essential amino acids (DMEM/FBS) (Life Technologies, Paisley, UK). Level of SPARC were analyzed by western blotting of cellular lysates and cell-conditioned media to confirm the efficiency of the silencing.

### Western blot analyses

After treatments, fibroblasts were lysed using 2x laemmli SDS sample buffer and separated by SDS-PAGE. Western blotting of cellular lysates was performed for β-actin (1:20,000, Sigma-Aldrich, Poole, UK), E-cadherin (1:500, Cell Signaling Technology, London, UK), Phospho-S522-β-catenin (1:500, Cell Signaling Technology, London, UK), β-catenin (1:500, Cell Signaling Technology, London, UK), Phospho-Y397-focal-adhesion kinase (1:250, Millipore UK Limited, Watford, UK), and focal-adhesion kinase (1:250, Millipore UK Limited, Watford, UK). Western blotting of cellular lysates and cell-conditioned media was performed for SPARC (1:500 Santa Cruz Biotechnology, Inc., Heidelberg, Germany), pan-histone H3 (1:1000, Millipore UK Limited, Watford, UK), and α-SMA (1:1.000, Sigma-Aldrich, Poole, UK). Immunodetected proteins were identified using the enhanced chemiluminescence system (Clarity Max Western ECL Substrate, Bio-Rad Laboratories Ltd, Watford, UK).

### Immunofluorescence staining

AECs were grown in monoculture using 8-well plates or in co-culture with fibroblasts using Transwells. At the end of each experiment, cells were fixed with 4% paraformaldehyde followed by permeabilization and staining with primary antibodies for Paxillin (Abcam 1:100), ZO-1 (Life Technologies 1:100), E-cadherin (Cell Signalling 1:100), and β-catenin (Cell Signalling 1:100). The secondary antibodies used were Alexafluor 488, 555, and 647 (all from BioLegend, London, UK). Cellular F-actin was stained using TRICT-phalloidin (Millipore UK Limited, Watford, UK). Cell nuclei were counterstained with 4′,6-diamidino-2-phenylindole, dihydrochloride (DAPI) 1:1000 dilution (Millipore UK Limited, Watford, UK). Cells were imaged using an inverted fluorescence microscope (Leica DMI 6000B, Leica Microsystems, Milton Keynes, UK) or an inverted confocal microscope (Leica TCS-SP5 Confocal Microscope, Leica Microsystems, Milton Keynes, UK).

### Statistics

All the experiments were performed at least in duplicate and repeated using at least two different donors. Results are expressed as means ± SD. Differences between groups were assessed using a Mann Whitney test. All data were analyzed using Prism (GraphPad, CA, USA). *p* < 0.05 was accepted as statistically significant. *p < 0.05, **p < 0.01, ***p < 0.001, ****p < 0.0001.

## Supplementary information

Supplementary figure legends

Supplementary figure 1 (S1)

Supplementary figure 2 (S2)

Supplementary Table 1
