# Supplementary material for: Paracrine SPARC signaling dysregulates alveolar epithelial barrier integrity and function in lung fibrosis
Source: Cell Death Discov. 2020 Jun 30;6:54. doi: 10.1038/s41420-020-0289-9 (PMC7327077; doi:10.1038/s41420-020-0289-9)
Supplement: Supplementary file 1 — Supplementary figure legends [file 41420_2020_289_MOESM1_ESM.docx]

**Fig.1S: Immunoblot of cell lysate and conditioned media from lung fibroblasts. A)** SPARC expression in conditioned media of IPFFs compared to NHLFs in absence or presence of TGFβ1. **B)** Protein expression cell lysate and conditioned media of IPFFs during SPARC silencing. **C)** Western blotting analysis of SPARC protein levels in CM from H441 lung epithelial cells (lane1), 3 different primary AECs donors (lanes 2-4) and 3 different primary IPFFs donors (lanes 5-7).

**Fig.2S: Primary AECs cell count in transwell co-culture with primary lung fibroblasts.** Cell count analysis of primary AECs in transwell mono-culture or in co-culture with IPFFs compare with NHLFs.

**Table S1:** List of the most abundant proteins detected in the conditioned media of IPFFs (in descending order of abundance).
