# Supplementary figures and images for: Paracrine SPARC signaling dysregulates alveolar epithelial barrier integrity and function in lung fibrosis
Source: Cell Death Discov. 2020 Jun 30;6:54. doi: 10.1038/s41420-020-0289-9 (PMC7327077; doi:10.1038/s41420-020-0289-9)

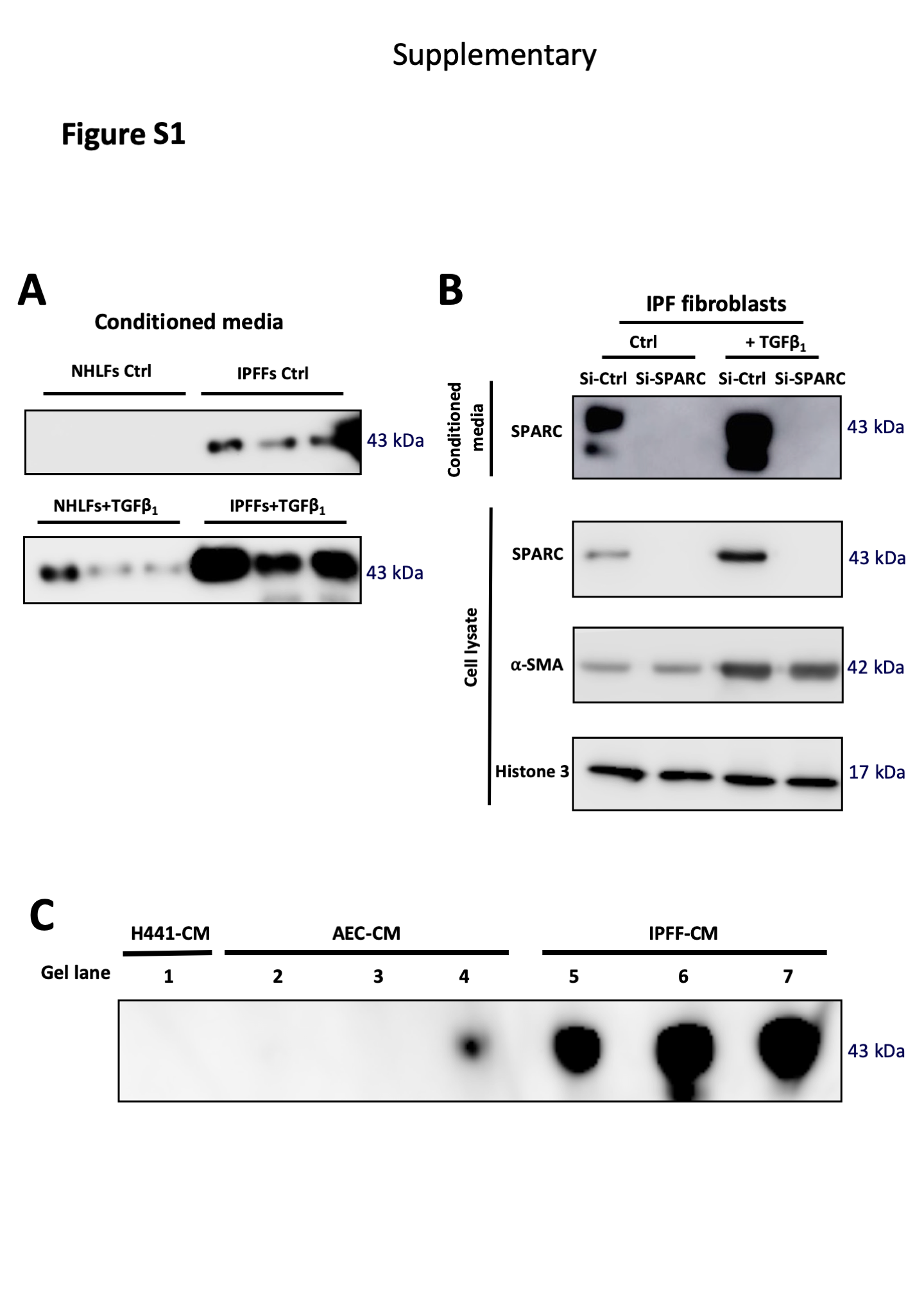

Supplement: Supplementary file 2 — Supplementary figure 1 (S1) [file 41420_2020_289_MOESM2_ESM.tif]

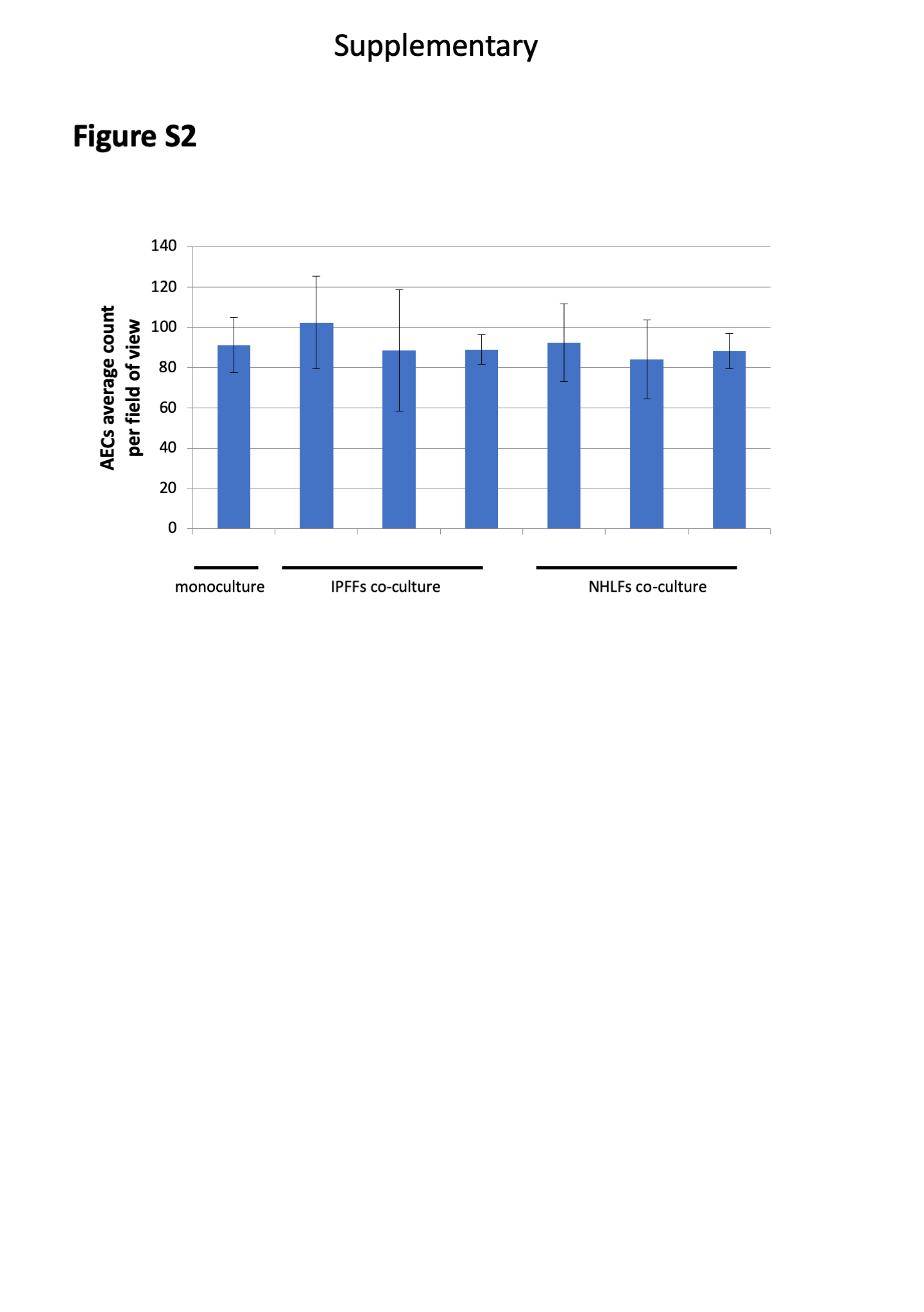

Supplement: Supplementary file 3 — Supplementary figure 2 (S2) [file 41420_2020_289_MOESM3_ESM.tif]
