# Supplementary material for: Paracrine SPARC signaling dysregulates alveolar epithelial barrier integrity and function in lung fibrosis
Source: Cell Death Discov. 2020 Jun 30;6:54. doi: 10.1038/s41420-020-0289-9 (PMC7327077; doi:10.1038/s41420-020-0289-9)
Supplement: Supplementary file 4 — Supplementary Table 1 [file 41420_2020_289_MOESM4_ESM.docx]

|  | Protein description |
| --- | --- |
| P02751 | Fibronectin OS=Homo sapiens GN=FN1 PE=1 SV=4 |
| P10909 | Clusterin OS=Homo sapiens GN=CLU PE=1 SV=1 |
| P08123 | Collagen alpha-2(I) chain OS=Homo sapiens GN=COL1A2 PE=1 SV=7 |
| P02452 | Collagen alpha-1(I) chain OS=Homo sapiens GN=COL1A1 PE=1 SV=5 |
| P07585 | Decorin OS=Homo sapiens GN=DCN PE=1 SV=1 |
| O00391 | Sulfhydryl oxidase 1 OS=Homo sapiens GN=QSOX1 PE=1 SV=3 |
| P60709 | Actin_ cytoplasmic 1 OS=Homo sapiens GN=ACTB PE=1 SV=1 |
| P12109 | Collagen alpha-1(VI) chain OS=Homo sapiens GN=COL6A1 PE=1 SV=3 |
| P08253 | 72 kDa type IV collagenase OS=Homo sapiens GN=MMP2 PE=1 SV=2 |
| P01033 | Metalloproteinase inhibitor 1 OS=Homo sapiens GN=TIMP1 PE=1 SV=1 |
| P24821 | Tenascin OS=Homo sapiens GN=TNC PE=1 SV=3 |
| Q15582 | Transforming growth factor-beta-induced protein ig-h3 OS=Homo sapiens GN=TGFBI PE=1 SV=1 |
| Q8TD10 | Mirror-image polydactyly gene 1 protein OS=Homo sapiens GN=MIPOL1 PE=2 SV=1 |
| Q8N4C6 | Ninein OS=Homo sapiens GN=NIN PE=1 SV=4 |
| P06681 | Complement C2 OS=Homo sapiens GN=C2 PE=1 SV=2 |
| P00924 | Enolase 1 OS=Saccharomyces cerevisiae (strain ATCC 204508 / S288c) GN=ENO1 PE=1 SV=3 |
| P51884 | Lumican OS=Homo sapiens GN=LUM PE=1 SV=2 |
| Q99715 | Collagen alpha-1(XII) chain OS=Homo sapiens GN=COL12A1 PE=1 SV=2 |
| Q16270 | Insulin-like growth factor-binding protein 7 OS=Homo sapiens GN=IGFBP7 PE=1 SV=1 |
| P00736 | Complement C1r subcomponent OS=Homo sapiens GN=C1R PE=1 SV=2 |
| P09871 | Complement C1s subcomponent OS=Homo sapiens GN=C1S PE=1 SV=1 |
| P12111 | Collagen alpha-3(VI) chain OS=Homo sapiens GN=COL6A3 PE=1 SV=5 |
| P02461 | Collagen alpha-1(III) chain OS=Homo sapiens GN=COL3A1 PE=1 SV=4 |
| P24593 | Insulin-like growth factor-binding protein 5 OS=Homo sapiens GN=IGFBP5 PE=1 SV=1 |
| P08670 | Vimentin OS=Homo sapiens GN=VIM PE=1 SV=4 |
| P26022 | Pentraxin-related protein PTX3 OS=Homo sapiens GN=PTX3 PE=1 SV=3 |
| Q12841 | Follistatin-related protein 1 OS=Homo sapiens GN=FSTL1 PE=1 SV=1 |
| P12110 | Collagen alpha-2(VI) chain OS=Homo sapiens GN=COL6A2 PE=1 SV=4 |
| P0C875 | Protein FAM228B OS=Homo sapiens GN=FAM228B PE=2 SV=1 |
| Q6PIY5 | Uncharacterized protein C1orf228 OS=Homo sapiens GN=C1orf228 PE=2 SV=2 |
| P05121 | Plasminogen activator inhibitor 1 OS=Homo sapiens GN=SERPINE1 PE=1 SV=1 |
| O43707 | Alpha-actinin-4 OS=Homo sapiens GN=ACTN4 PE=1 SV=2 |
| P01034 | Cystatin-C OS=Homo sapiens GN=CST3 PE=1 SV=1 |
| Q4L180 | Filamin A-interacting protein 1-like OS=Homo sapiens GN=FILIP1L PE=1 SV=2 |
| P09486 | SPARC OS=Homo sapiens GN=SPARC PE=1 SV=1 |
| Q7Z7A1 | Centriolin OS=Homo sapiens GN=CNTRL PE=1 SV=2 |
| A8MPP1 | Putative ATP-dependent RNA helicase DDX11-like protein 8 OS=Homo sapiens GN=DDX11L8 PE=1 SV=1 |
| P07093 | Glia-derived nexin OS=Homo sapiens GN=SERPINE2 PE=1 SV=1 |
| O95613 | Pericentrin OS=Homo sapiens GN=PCNT PE=1 SV=4 |
| P68032 | Actin_ alpha cardiac muscle 1 OS=Homo sapiens GN=ACTC1 PE=1 SV=1 |

**Table S1: List of the most abundant proteins detected in the conditioned media of IPFFs (in descending order of abundance).**

Supplementary
